# Role of interventional radiology in line insertion on intensive care during the Covid-19 pandemic

**DOI:** 10.1186/s42155-020-00171-w

**Published:** 2020-10-20

**Authors:** Tom Cavenagh, Sofia Katsari, Bhavin Kawa, Raham Karimaghaei, Vyzantios Pavlidis, Vasileios Patsiogiannis, Nikolaos Ntagiantas, Joo-Young Chun, Seyed Renani, Leto Mailli, Michael Gonsalves, Lakshmi Ratnam, Raj Das, Robert Morgan

**Affiliations:** Interventional Radiology, St George’s Healthcare NHS Foundation Trust, Blackshaw Road, Tooting, London, SW17 0QT UK

We write to inform the readership of the beneficial role of interventional radiology (IR) in providing an urgent central venous access service to patients in the intensive care units (ICU) of our hospital during the peak surge of the current Covid-19 pandemic.

The World Health Organisation declared the Covid-19 outbreak a global pandemic on March 11th, 2020. Among several measures that were taken by the United Kingdom’s National Health Service was a significant increase in ICU capacity. Our own institution increased ICU beds from 65 to 155. As was the case in many IR departments (Morgan et al., 2020; Rostampour et al., 2020), to assist with the increased demand, we volunteered to insert the central venous and peripheral arterial lines on the ICU in “Lines Teams” during peak ICU usage. Here we share our experience and audit the outcomes of this intervention.

The lines team operated from the 1st-24th April and consisted of one IR consultant and fellow providing an out-reach service between 8 am-6 pm. Individuals would rotate daily. During the Covid-19 outbreak we continued all urgent and emergency work, also covered by a single consultant and fellow. IRs not rostered would remain available in case of sickness or requirement to self-isolate. It should be noted vascular surgery also provided a lines service.

In line with national guidance (Gov.uk coronavirus (COVID-19): guidance and support, 2020), we wore full personal protective equipment during line insertion as the ICU was categorised as a high-risk environment. If one unit required multiple lines, we would remain in full PPE between patients but don a new sterile outer gown and gloves. Initially all equipment would be collected from the ICUs. However, considerable time was spent familiarising with the different units, therefore, from a checklist, our IR nurses created ‘grab bags’ to take from IR. Portable ultrasound scanners (USS) were available on the ICU. However, fluoroscopy was not. It is well documented that image-guidance reduces complication rates (Kornbau et al., 2015). In particular, USS reduces the risk of inadvertent arterial puncture, and fluoroscopy the risk of catheter misplacement and venous laceration during dilatation. Anecdotally, we did not find the lack of fluoroscopy too bothersome.

To audit our practice, data was collected retrospectively between April 1st-24th 2020 using the Integrated Clinical Information Programme and Picture Archiving and Communication system. All lines inserted by IR were included. Data collected included line type, insertion site, technical success, and immediate complications (arterial puncture, haematoma, vascular laceration). When applicable, post-procedural chest radiographs were reviewed for adequate positioning, pneumothorax and haemothorax.

Approximately 120 lines were inserted on Covid ICUs during the audit period, of which, IR inserted 45 lines during 35 patient encounters. Vascular surgery would have inserted a proportion of the remaining lines, however, their data is not presented here. Line type and location were: 29 quad-lumen central venous lines (internal jugular vein; right:11, left:17, right common femoral vein:1); 13 non-tunnelled dialysis catheters (internal jugular vein; right:6, left:6, right common femoral vein:1); and 3 radial arterial lines. Peak referrals reached 6 patients/7 lines in 24 h. No immediate complications were reported (0%). Post-procedural radiographs revealed no instance of pneumothorax/haemothorax (0%). One left-sided central venous line tip was malpositioned in the left brachiocephalic vein (2.5%). Reported complication rates for line insertion in the literature are, 3–32% tip malposition without imaging guidance and 0–4% with imaging guidance (McBride et al., 1997), 1–2% pneumothorax/arterial puncture and less than 1% great vessel perforation (Funaki, 2002). Our outcomes are, therefore, better than those reported in the literature for non-image-guided insertion and in line with image-guided insertion.

In summary, implementation of the ‘lines team’ was a successful and well utilised resource during the peak of the Covid-19 outbreak. This illustrates the versatile nature of IR, which in crisis situations can play a significant role in the delivery of aspects of critical care.

## Data Availability

The datasets used and/or analysed during the current study are available from the corresponding author on reasonable request.
